# Let’s Talk about TEX—Understanding Consumer Preferences for Smart Interactive Textile Products Using a Conjoint Analysis Approach

**DOI:** 10.3390/s18093152

**Published:** 2018-09-18

**Authors:** Julia Offermann-van Heek, Philipp Brauner, Martina Ziefle

**Affiliations:** Human-Computer Interaction Center, RWTH Aachen University; Campus Boulevard 57, 52074 Aachen, Germany; brauner@comm.rwth-aachen.de (P.B.); ziefle@comm.rwth-aachen.de (M.Z.)

**Keywords:** smart interactive textile products, technology acceptance, consumer preferences, conjoint analysis, user segments, smart chair, smart jacket

## Abstract

Interactive textiles are reaching maturity. First technology augmented textiles in form of clothes and furnitures are becoming commercially available. In contrast to the close link between technological development and innovations, future users’ acceptance and usage of such interactive textiles has not been integrated sufficiently, yet. The current study investigates future users’ consumer behavior and acceptance of interactive textiles using a scenario-based conjoint analysis study, which was presented in an online questionnaire (n=324). Two prototypical interactive textiles were focused on: a smart jacket and a smart armchair. To assess the textile products, the participants had to choose the preferred product alternative consisting each of the acceptance-relevant factors “connectivity”, “input modality”, “feature range”, “usability”, and “ease of cleaning”and their respective levels. The results revealed that the “ease of cleaning” is the most important decision criterion for both textile devices (even more important for the smart jacket), followed by “feature range”, “connectivity”, and “usability”. In contrast, the “input modality” is perceived as least important. The study also identified user profiles based on the projected consumer behavior (“adopters”, “rejecters”, and “undecided”) for both products. Besides the differences in product evaluation and projected consumer behavior, the user groups are significantly influenced by the individual affinity to textiles (both products) and gender (smart jacket). The findings are used to derive design and communication guidelines referring to interactive textiles in order to incorporate users’ needs, wishes, and requirements into future products.

## 1. Introduction

Moore’s law predicts that complexity of integrated circuits and computing technology grows exponentially [1]. While availability, potential, and performance increase, technology shrinks in power consumption, prize, and size. This development leads to a growing integration of computing technology in a vast amount of everyday objects. Many researchers have envisioned how this will affect work and private environments. In Xerox PARC (Palo Alto, California, USA), for example, Weiser and his team contemplate how office environments will change if computing technology and connectivity are available in smart white-boards, desks, or lamps and they coined the term “ubiquitous computing” for technology that is integrated into everyday objects [2]. “Ambient Intelligence” [3,4] refers to a similar concept where computing technology fades into the environment and intuitively offers information and support. These once bold visions are now readily available: smart light and heating react to peoples’ presence rather than static schedules, or personal drones in form of robotic vacuum cleaners start cleaning when nobody’s home.

One of the next frontiers of this technical development is the integration of computing technology, connectivity, sensors, and actuators in fabrics and textile products, which would unleash its potential from its conventional covers [5]: plastic, metal, or glass. This development is especially challenging, as the history of humanity is deeply interwoven with the history of textiles. Early traces to the use of textiles by humans date back to 30.000 B.C. [6,7]. Textiles are perceived as warm, soft, and fashionable and come in an extensive variety of different forms, colors, surfaces, and sizes. Consequently, the key challenge for research and design is to integrate the rather novel idea of computing technology with textiles, that are so deeply rooted in our history, to reduce potential gaps in social acceptance, and to harmonize the interaction between us humans and the technology.

As key contributions of this work, the conjoint study presented in the following:

- Gives insights in users’ weighting of decision-relevant acceptance factors referring to two prototypical interactive textile products,
- Identifies user segments differing in their decision and acceptance behavior influenced by user diversity,
- Derives user segment-tailored design and communication guidelines for interactive textiles.

The remainder of this article is structured as follows: first, we present the current state of the art on the technical development of smart interactive textiles, methods to assess the acceptance of products in advance, and outcomes of studies on the acceptance and use of textile interfaces in Section 2. Next, we introduce the applied conjoint analysis approach in Section 3. Afterwards, we present the results for the whole sample, followed by results focusing on specific user segments in Section 4. Section 5 discusses the results of the study and presents suggestions and guidelines for designing accepted and usable smart interactive textiles. Finally, Section 6 concludes this article summarizing the main findings and contributions of the undertaken study.

## 2. Related Work

This section presents the theoretical background of the study focusing on the current state of the art with respect to smart and interactive textiles, technology acceptance, and acceptance of interactive textiles.

### 2.1. Smart and Interactive Textiles

The idea of integrating electronics into textiles emerged over twenty years ago: Post et al. augmented fabrics with sensors, actuators, power distribution, and supply as well as with communication interfaces [8,9].

The main challenges for designing interactive textiles stem from three areas: First, the identification or development of sensors, actuators, and microprocessors that can be adapted or integrated into textiles and fabrics. Second, the development of new textile devices and interfaces that use the technologies as well as the design of gestures and new interaction paradigms to harness the additional affordances of textile surfaces. Third, the integration of the users’ perspective plays a decisive role in order to be able to design user-oriented and well-accepted interactive textiles.

With regard to the first challenge (technical perspective), recent overview articles [5,10,11] on technical foundations of interactive textiles show that this area is intensively investigated and research on smaller, more durable, and more precise sensors embedded in textile devices is ongoing. The review article from Castano and Flatau concludes that complete smart fabric systems are enabled by the integration of textile-based functional elements [11]. Recently, Poupyrev et al. showed that interactive textiles are no longer limited to hand-made prototypes by integrating conductive yarns into textile fabrics by industrial looms, which enables manufacturing of interactive textiles at scale [12].

Concerning the second challenge (devices and interaction paradigms), recent research has shown the huge potential of interactive textiles. For example, Rekimoto et al. showed that textile surfaces offer new interaction paradigms that can harness the affordances of textile surfaces, folding, bending, or stretching [13]. Karrer et al. used the deformability of fabrics as an input device. They developed a textile that detects pinching and rolling of a textile fold with the fingers. This technology might be integrated into cloths and can then be used for eyes-free control of, e.g., a media player [14,15]. This concept was extended by Hamdan et al. to a two-dimensional textile surface that can be grabbed and interacted with [16]. In addition, Saponas et al.’s presented PocketTouch, a system that enables gesture recognition on flat surfaces in wearable textiles [17]. In regard to textile interfaces in non-wearable contexts, Rus et al. built a couch that facilitates touch interaction through capacitive sensing integrated into its textile surface [18].

In contrast to the first and second challenge, the integration of the users’ perspective (third challenge) is often missing, as users are often not integrated in the development process of innovative products. Thus, their perspectives, wishes, and requirements are neither contributing, nor steering the development process, which may yield products with unnecessary low ease of use, low usefulness, or low acceptance.

### 2.2. Technology Acceptance

The understanding of the requirements of societal adoption of new technologies can be approached from many perspectives. Roger’s theory of the *Diffusion of Innovations* [19] directs to an explanation of why and when innovations are adopted. The theory describes five stages of technology adoption in line with five distinct user personalities, that differ in regard to the time of the adoption. In regard to the different personalities, Rogers differentiates between “innovators”, “early adopters”, “early majority”, *late majority*, and *laggards*. Following Rogers, the adoption of new technology goes through different distinct phases: First, a new technology becomes known. Then, positive and negative attitudes towards the technology are formed. After implementation and usage of the technology, a re-evaluation takes place in the feedback phase. Based on Rogers, it is recommended to integrate the users’ perspective as early as possible in the development process of innovative products. This suggests that even product ideas and concepts should be evaluated by participants, e.g., in a scenario-based approach, in order to satisfy users’ requirements and needs and to integrate them into the product development process.

Another perspective and access to technology acceptance is the quantitative Technology Acceptance Model (TAM) by Davis aiming for a prediction of system and user factors that relate to a successful technology adoption [20]. It is grounded in Fishbein and Ajzen’s Theory Of Reasoned Action (TRA) [21] and Theory of Planned Behavior (TPB) [22]. The first theory postulates that the actual behavior—in our case the usage of a technology—is governed by the individual intention to perform the behavior and that this intention is controlled by the attitude towards the behavior and subjective norms. The latter is a refinement that includes the personal self-efficacy believes as an additional explaining factor into the model. The TAM showed that the actual use of office software is predominantly predictable by the intention to use the software, which in turn is governed by the attitude towards using it, the perceived usefulness, and also the perceived ease of use. Summarizing, the behavioral intention to use products or technologies can be used as an indicator for the acceptance and adoption of innovative technologies and products.

The traditional models of technology acceptance (TAM [20], Unified Theory of Acceptance and Use of Technology (UTAUT2) [23]) have in common that they provide insights into the perception of single acceptance-relevant criteria of a technical scenario or product. However, in real life scenarios, users do not evaluate single product characteristics separately from each other, but rather weigh positive connoted aspects against less positive or negative aspects in order to come to the decision to use or drop a technology innovation. Thus, the acceptance models do not allow to holistically simulate and portray complex decision scenarios, in which several decision criteria are weighted against each other. Hence, another methodological approach is needed to investigate the relationship of decision-relevant factors for interactive textiles’ acceptance (see Section 3).

### 2.3. Technology Acceptance of Interactive Textiles

Due to the novelty of smart interactive textiles, the research landscape on the acceptance of smart interactive textiles still has many blind spots. Perry [24] studied the consumer’s needs and purposes for smart clothing. In general, the study revealed a high consumer interest in smart clothing, but the study also revealed conflicting design goals: For designers, it is most important that smart clothes are affordable, fashionable, and enjoyable, whereas they believe consumers to consider function, no technical problems, and affordability as the most important criteria. Hence, the study showed that designers do not fully understand consumer needs. To inform designers, there is a need for an empirical description of consumers’ requirements of smart textiles. To satisfy the requirements and wishes of future users adequately, future users have to be directly integrated in design and evaluation processes of smart textiles. In this regard, only a few studies have considered smart textiles’ acceptance, its projected use, and its determining factors so far.

For example, van Heek et al. contrasted product properties that relate to product acceptance of a smart shirt for recreational and medical use [25]. The study showed that the usage context influences the overall acceptance. Also, it was shown that users perceive a smart textile product’s washability as crucial for acceptance.

In a recent study, Hildebrandt et al. analyzed the general design-space of textile products, without zooming into specific products or product scenarios [26]. As key result from analyzing the design space of smart textiles, it was found that users’ most important criterion is the textile integration, followed by the requirement that the technology should be unnoticeable and fade into the background.

Using a scenario-based approach Ziefle et al. gathered people’s requirements on interactive textiles for different contexts in the smart home and compared perceived benefits and perceived barriers [27]. The study revealed both, requirements that apply similarly across the different usage context (e.g., price, durability, and washability) as well as differences: For example, the integration of electronics into carpets was not rated as useful, whereas the usefulness of smart textiles for clothes and furnitures was rated as highest.

Based on the evaluation of a cushion with an interactive surface and building on established models to predict the acceptance of consumer technology, Brauner et al. derived a technology acceptance model specifically tailored to the specific characteristics of smart textiles (the Smart Textile Technology Acceptance Model, STTAM) [28]. The model shows that both individual user factors and product factors shape the projected acceptance of smart textiles. The most decisive product factors were the perceived ability to integrate the smart cushion seamlessly into ones daily routine, its hedonic value (fun to use) as well as the expected performance (what can be done with it). From the perspective of user diversity, a persons’ attitude towards technology influences the overall intention to use smart interactive textile devices.

Brauner et al. evaluated the key predictors for the acceptance of an interactive armchair (a motorized recliner armchair augmented with interactive textile surfaces) [29]. Users evaluated this interactive demonstrator in regard to its pragmatic and hedonic values, its attractiveness, and the overall acceptance (measured by the usage intention). Beyond the importance of the pragmatic values, key acceptance criteria were the hedonic properties (e.g., is the chair fun and pleasurable), and its overall attractiveness, in line with the invisible integration of the technology into the natural properties of the chair.

Those conducted studies in the area of smart textiles’ acceptance have in common that they evaluate relevant factors (e.g., requirements, benefits, barriers) separately and do not allow to draw conclusions about the relationships between decision-relevant factors to adopt a specific textile product.

### 2.4. Research Gaps and Research Aims

Overall, previous research on the acceptance of interactive textiles shows that smart textiles implemented in wearables or furnitures are generally evaluated positively. Yet, the research landscape has several research gaps that will be addressed in the following:

**Gap 1—Specificity of evaluation criteria:** It is not known yet which specific product properties are relevant for the overall acceptance and how these properties relate or compensate each other.

**Gap 2—Specificity of textile products:** It is unclear if consumer requirements for clothes (textiles to be worn close to the body) and textiles for furnitures (remote usage) are similar or, if different criteria are perceived as relevant.

**Gap 3—Specificity of consumer profiles:** It is not known if there are specific user groups which tend to adopt textile products and which evaluation criteria might account for the acceptance decision.

To address these gaps, this study

1. Evaluates different aspects of textile input devices (connectivity, usability, ease of cleaning, input modality, and feature range). To understand the relation among these evaluation criteria and the relative importance of the criteria for the acceptance decision, we use an experimental setting which allows determining their trade-offs.
2. Uses two interactive textile products for everyday use: clothes and furnitures. Both products are widely known and used, have thus a high reach for many consumers and allow a broad understanding of the acceptance and perceived usefulness of textile products. To understand individual acceptance patterns, all participants evaluated both product types (randomized order).
3. Explores user diversity in order to describe the commonalities and differences within and across different user types. On the base of the response patterns, user profiles were formed (by user segmentation procedures) and referred to demographics (age, gender) but also attitudinal factors (technical self-efficacy, affinity to textiles, and the experience with smart textiles).

With this structured empirical-experimental approach the current study (1) allows empirically uncovering consumers’ acceptance profiles for two everyday textile products; (2) clarifies the importance of the textile properties underlying the acceptance decisions; and (3) identifies consumer profiles for smart interactive textiles. The outcomes are not only relevant for technology acceptance research, but might be highly informative, on the one hand, for designers to develop user-centered technical products and, on the other hand, also for marketing experts to derive individually tailored information and communication strategies.

## 3. Method and Materials

This section presents the empirical approach of the study. First, the conjoint analysis approach is shortly outlined, followed by the selection of relevant attributes. Next, the experimental design of the online questionnaire is described. Finally, the applied statistical procedures as well as the study’s sample are presented.

### 3.1. Key Characteristics of the Conjoint Analysis Approach

Luce and Tukey developed the conjoint analysis (CA) approach as a quantitative empirical research method based on the combination of a measurement model and a statistical estimation algorithm [30]. The analysis and simulation of consumers’ preferences and decisions for innovative products represent the key features of CA. By combining diverse product properties (in the context of CA called attributes, e.g., the product color) and their respective characteristics (levels, e.g., green, yellow, blue) in a set of product scenarios, study participants and future consumers choose the product alternative they prefer the most. This way, a weighting of product properties, the investigation of trade-offs between different products properties and characteristics, as well as segmentations of user groups are enabled.

Initially, the CA approach was used mainly in economic science and market research enabling the assessment of innovative products and respective specificities. Since the last years, CA has been increasingly used within the landscape of acceptance research as well-covering diverse contexts, such as the acceptance of information systems [31], decisions within the context of healthcare [32,33], or acceptance studies focusing on energy technologies, e.g., [34,35].

The key advantage of CA—compared to conventional quantitative online questionnaire approaches—lies in the investigation of comparably complex decisions referring to new and innovative products: within these decisions, all attributes and levels of a product are directly weighted against each other [36,37] and are thus not evaluated separately.

As a further advantage, CA enables the analysis of users’ and consumers’ perceptions of future, new, innovative, or even scenario-based imaginary (and not yet on the market available) products. As a result of the participants’ assessment of a set of product alternatives (consisting of a defined number of attributes and differ from each other in the attribute levels), complex decision processes can be simulated revealing—after a decomposition—relative importances for the attributes and separate underlying part-worth utilities for the attribute levels [36,37]. The relative importance of an attribute shows which attribute influences the participants’ choice the most. Furthermore, the part-worth utilities indicate which attribute level is evaluated highest or lowest and consecutively contributes—in tendency—positively or negatively to the product decisions.

Conjoint analysis provides diverse approaches enabling different simulations and analyses. In this study, a choice-based-conjoint analysis approach (CBC) was applied, as it imitates decision processes with more than one attribute that probably influences the final product decisions [38]. During the CBC procedure, the participants are repeatedly asked to select the most preferred product alternative out of a set of product configurations.

### 3.2. Selection of the Relevant Attributes

Many evaluation criteria shape the adoption of novel information and communication technology in general and smart textile products in particular. Based on our prior research, an extensive literature review, and additional workshops with smart textile experts, we collected attributes which were identified as crucial for smart textiles. The following aspects were finally chosen as attributes for the quantitative conjoint analysis approach. Table 1 shows an overview of all attributes and the respective levels including their visual representations.

As “usability” and ease of using a product is often seen as a key constituent for the acceptance of a product or service [20], it is integrated as attribute in the conjoint analysis. In this study, we contrasted three different levels of usability of a product: “easy”, “middle”, and “difficult”. The first level “easy to use” refers to a product that is very easy or even intuitively to use and requires no learning effort. The second level “middle” refers to a product having a middle level of usability meaning that the users have to put an extra effort, e.g., reading a manual. However, once the usage principle is understood, the operation of the product is easy. The third level “difficult” refers to a product with a more difficult handling: even after repeated usage of the product, assistance, e.g., in the form of a manual, has to be consulted. To learn all functionalities requires a high additional effort.

For an integration of smart interactive textiles in the private home environment, the “connectivity”, i.e., connecting the smart interactive textiles with other systems (e.g., smartphone, network) is also a central issue [39]. Thus, enquoteconnectivity is integrated in the conjoint analysis including the levels “not connected”, “low (port)”, and “high (network)”. The first level “not connected” contains a product that can only be operated by itself and is not able to interact with other devices or systems. The second level “low (port)” refers to a textile product with a low connectivity meaning that it can be connected and can communicate via a port with a smart-phone, for instance. However, the number of interfaces and opportunities is limited. The third level “high (network)” contains a product with a high connectivity meaning that it can be integrated within a smart home system and is able to communicate with all devices belonging to the smart home system.

In preceding studies with both experts of smart interactive textiles as well as laypeople, *ease of cleaning* was perceived as an important criterion [25,28]. Thus, different levels of “ease of cleaning” are integrated in the current study: “usual cleaning”, “additional expense”, and “special cleaning”. The level “usual cleaning” refers to a product that can be cleaned like conventional textile products. An “additional expense” means that the product can be cleaned at home using a special component (e.g., special detergent), that has to be bought. “Special cleaning” means that the product cannot be cleaned at home, but needs to be taken to a special cleaning company.

A more technical issue that is discussed in the context of smart interactive textiles refers to the “input modality”, i.e., the way the product can be operated (e.g., gestures or voice control [40]). In this study, three different types of “input modalities” are integrated: “simple”, “extended”, and “complex”. A “simple” input modality means that the product can be operated by direct gestures on the textile surface (e.g., swiping, touching, pinching). A product with an “extended” input modality has a second input modality (e.g., voice or smart-phone input). A product with a “complex” input modality contains diverse operating options (e.g., a combination of gesture, voice, and smart-phone).

A last aspect refers to the “feature range” of smart interactive textile asking for what makes a product “smart” or what does a “smart product” do [41]. Thus, the “feature range” is also integrated as an attribute in the conjoint analysis including the levels “conventional”, “extended”, and “complex”. A “conventional” feature range means that the product has the same functions like textile everyday products. An “extended” feature range refers to a product enabling interactive functions (e.g., to accepting a call (jacket) or operating seating positions (armchair)). A “complex” feature range contains—besides the functions of the extended range - also more complex operating options such as operating home automation, light, or music.

### 3.3. Experimental Design

Five attributes (“Connectivity”, “Ease of Cleaning”, “Input Modality”, “Feature Range”, and “Usability”) with each three levels were chosen for the CBC approach and the participants performed the conjoint analysis for two different smart interactive textile products: a “smart jacket” and a “smart armchair”. All respondents evaluated both products (repeated measures design) in a randomized order.

In the CBC tasks for the smart jacket and the smart armchair, each time three sets of product configurations were presented and a forced-choice response format was chosen (no “none-option” was included; see Appendix A for an illustration of a decision task). All attribute levels and their graphic representations were introduced before the selection tasks, followed by short explanations of each attribute and all respective attribute levels. Before the study was started, the complete questionnaire including the visualization of attributes and levels as well as introductory texts were checked and pretested several times with regard to comprehensibility, legibility, and clearness.

As a further feature, CA allows the reduction of stimuli which are presented to the participants, as—in the present study—a full-factorial combination of all corresponding levels would have led to 243 (3×3×3×3×3) possible combinations, each for the smart jacket and the smart armchair. Therefore, the respondents completed seven random choice tasks in each study (jacket and armchair). This reduction may have led to the fact that some levels of attributes might not appear together in one set and the participants most probably do not evaluate the same product configurations. To ensure that the design was comparable to the hypothetical orthogonal design, we tested the design efficiency and the test confirmed a median efficiency of 99% relative to a hypothetical orthogonal design. As a further efficiency criteria, the design’s standard error was below the maximal valid limit of 0.05 for both studies [42] and, thus, confirmed the design efficiency as well.

### 3.4. Structure of Online Questionnaire

**Figure 1:**
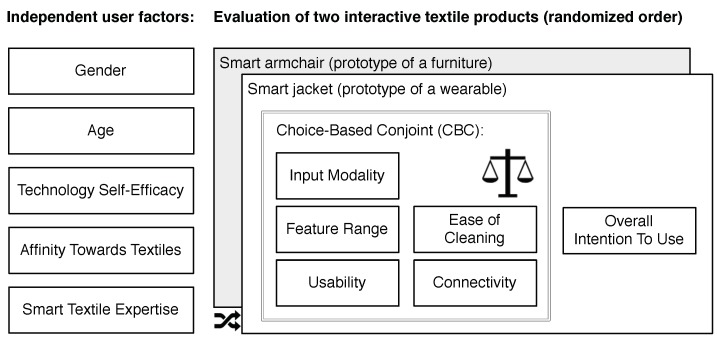
Design of the survey to study the individual weightings of product properties for two different smart textile products.

TThe structure of the online questionnaire is illustrated in Figure 1. In the first part of the online questionnaire, the participants were asked for demographic information namely *Gender*, *Age*, and level of *education*. In the next part, we assessed the participants’ attitudes towards *self-efficacy in interacting with technology (SET)* using 4 items (α=0.883) based on [43] (e.g., “I really enjoy cracking a technical problem“), *affinity towards textiles* using 5 items (α=0.708) (e.g., “I love owning new textiles“) (see Table A1), as well as their *experience with smart textiles* using 5 items (α=0.883) (e.g., “I have already heard the term Smart Textiles“).

Afterwards, the selected attributes and levels of the conjoint analysis’ choice tasks were introduced. Subsequently, the smart armchair and the smart jacket were introduced and each evaluated with 7 random choice tasks (consisting of combinations of all attributes and levels (see Section 3.2)) in a randomized order. In each choice task, the participants were asked to choose the product configuration that meets their wishes and needs best. A typical example of a choice task—the participants performed—is presented in the Appendix (see Appendix A).

Following the conjoint choice tasks, the participants assessed their acceptance to use a smart jacket (α=0.926) and a smart armchair (α=0.893) using three items that captured the intention to use from Davis’ Technology Acceptance Model and subsequent models.

All items (attitudinal characteristics and intention to use) were evaluated using six-point Likert scales (0 = strongly disagree; 5 = strongly agree). Values > 2.5 indicated approval, while values < 2.5 indicated rejection. Finally, the participants provided feedback and comments on the topic and the questionnaire on an optional basis.

### 3.5. Data Collection and Description of the Sample

The invitation to participate in the study was distributed personally, via email, and by technology-mediated social networks. In total, 584 participants took part in the study. As only complete data sets could be used for further analyses, a sample of *n* = 324 data sets remained. Within the sample, *gender* was equally spread with 49.4% males and 50.6% females. The mean *age* of the participants was 26.9 years (±9.1; min=15; max=60). The *educational level* of the participants was high with 33.3% holding a university degree and 42.6% an university entrance certificate. Concerning attitudinal characteristics, the participants reported on average a high *self-efficacy in interacting with technology* (M=3.76±1.05; min=0; max=5), a rather neutral *affinity to textiles* (M=2.61±1.04; min=0; max=5), and, overall, a low *experience with smart textiles* (M=2.17±0.97; min=0; max=5).

An initial correlation analysis revealed the following relationships between user factors and the perceived ease of use as well as intention to use both smart interactive textile products independent from the conjoint analysis results. Neither age, nor educational level were related with the perceived ease of use nor with the intention to use both products. Gender was also not related with the perceived ease of use both textile products. However, the intention to use the smart jacket was slightly related with gender, revealing men to be more likely to use the smart jacket than women (r=−0.133;p<0.05). With regard to the attitudinal characteristics, more significant relationships were found: self-efficacy in interacting with technology correlated significantly with perceived ease of use the smart jacket (r=0.247;p<0.01) and the smart armchair (r=0.179;p<0.01). Furthermore, there were slight tendencies of relationships between the smart textile expertise and the intention to use the smart jacket (r=0.135;p<0.05) as well as the smart armchair (r=0.129;p<0.05). Even stronger correlations were found for the affinity towards textiles and the intention to use the smart jacket (r=0.208;p<0.05) and the smart armchair (r=0.332;p<0.05). These first correlation results suggest to analyze the influence of user factors on the participants’ decisions in detail (see Section 4.3).

### 3.6. Data Analysis

We used Sawtooth Software for the analysis of the decision tasks [44]: First, we calculated the relative importance scores of the five attributes delivering information about how important an attribute is for the product selection relatively to all other attributes. Second, the part-worth utility values were calculated and part-worth utility scores were deduced indicating which attribute level—in relation—contributes positively or negatively to the product selection [36,44]. Finally, different user segments were identified using Latent class segmentation analysis (LCA). Besides conjoint-related procedures, data was analyzed using descriptive as well as inference statistical analyses. Prior to these analyses, we checked measurement quality. A Cronbach’s α>0.7 indicated a satisfying internal consistency of the scales. With respect to the description of user segments and their respective evaluations, data was analyzed by correlation and (M)ANOVA (multivariate analysis of variance)procedures. The type I error rate (level of significance) was set at 5%.

## 4. Results

This section presents the results of our study starting with the relative importance of attributes for the choice tasks related to the smart jacket as well as the smart armchair, followed by the description of the respective meanings (part-worth utilities) of the attribute levels are described. Finally, the results of a user group segmentation are detailed. A summarizing overview of all descriptive and inference statistic data is presented in different Tables in the Appendix. Table A2 shows the results for the whole sample comparing the smart jacket and the smart armchair. Table A3 presents the user segment results for the smart armchair and Table A4 for the smart jacket.

### 4.1. Relative Importance Scores

**Figure 2:**
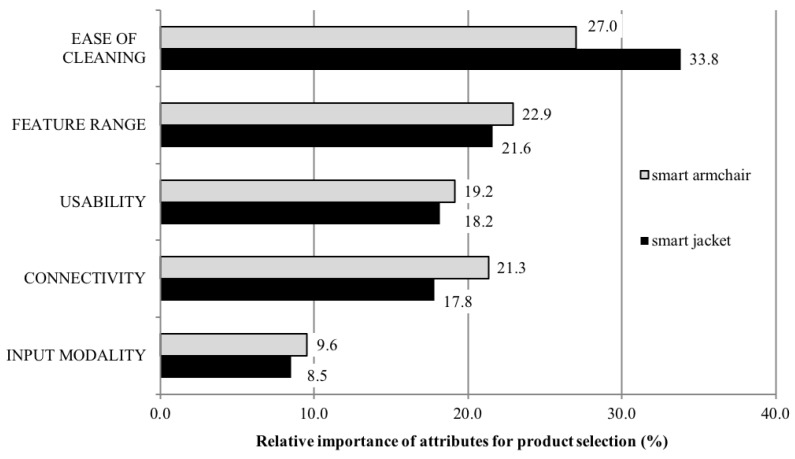
Relative importance of attributes for smart jacket and armchair.

First the relative importances of the product attributes of the two products are reported (Figure 2). “ease of cleaning” was the most important factor influencing the participants choice for both textile products, however, it was significantly more important (F(1323)=83.488; p<0.01) for the smart jacket (33.83±13.12%) compared to the smart armchair (27.01±12.49%). “Feature Range” was the second most important factor that influenced the acceptance of the smart armchair (22.95±10.50%) slightly more (F(1323)=3.592;p=0.059, n.s.) than acceptance of the smart jacket (21.62±11.82%). The attribute “Connectivity” was ranked third and was significantly more important (F(1323)=28.078; p<0.01) for the smart armchair (21.33±10.25%) than for the smart jacket (17.83±11.72%). The fourth most important factor is the “Usability” of the devices. It was almost equally relevant (F(1323)=3.168; p=0.076, n.s.) for the acceptance of the smart jacket (18.20±7.83%) and the smart armchair (19.17±9.62%). In contrast, “Input Modality” was the least important factor influencing the acceptance for the smart jacket (8.51±5.61%) as well as the smart armchair (9.55±6.27%)(F(1323)=6.715; p<0.05).

It should be noted that the order of the relative importances was the same for both products and only differs for the attributes “Usability” and “Connectivity”. For the smart armchair, usability was considered as less important than its connectivity, whereas the usability and connectivity were evaluated as equally important for the smart jacket.

### 4.2. Part-Worth Utilities: Meaning of Attribute Levels

**Figure 3:**
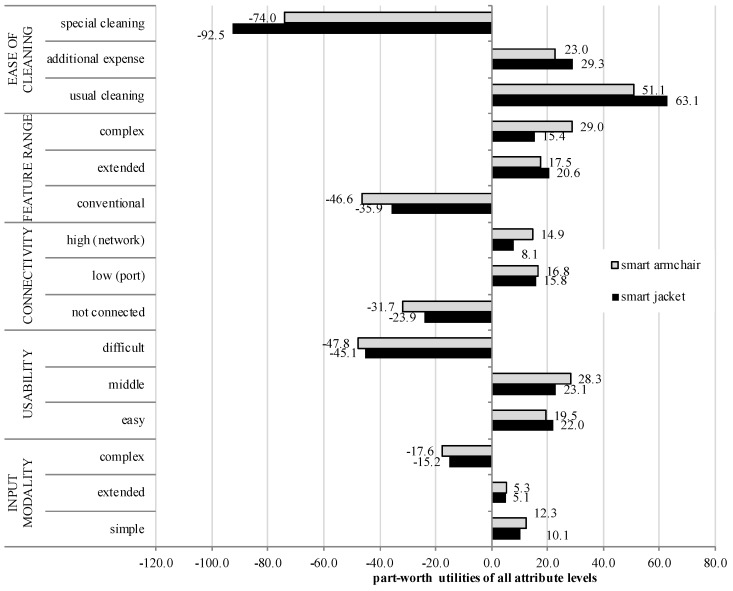
Part-worth utilities of all attribute levels for the smart jacket and the smart armchair.

In Figure 3, the average zero-centered part-worth utilities are shown for all attribute levels and for both textile products. Based on the part-worth utilities, attribute levels with the highest positive and negative evaluation and thus, product configurations with the highest and lowest potential of acceptance can be identified.

For both products, the best product configuration did not differ strongly: it contained of the characteristics “usual cleaning”, a “middle usability”, a “low (port) connectivity”, and a “simple input modality”. The only difference concerning the best product configuration referred to the attribute “Feature Range”: here, the best configuration was a complex feature range for the smart armchair (i.e., attribute with highest complexity), while it was the extended feature range for the smart jacket (i.e., attribute with medium complexity). The evaluations of both products did also not differ with regard to the worst product configuration consisting of the attribute levels “special cleaning”, “difficult usability”, “conventional feature range”, “not connected”, and “complex input modality”.

Correspondingly to the high relative importance of the attribute “ease of cleaning” for both products, it also showed the largest span of part-worth utilities for both products, while “usual cleaning” (chair: +51.1; jacket: +63.2) and “additional expense” (chair: +23.0; jacket: +29.3) in tendency contributed positively to the decisions. In contrast, “special cleaning” (chair: −74.0; jacket: −92.5) had a clear negative influence on the decision for a product alternative.

Looking at “Connectivity”, the level “not connected” was rejected for both products (chair: −31.7; jacket: −23.9). In contrast, a “high (network) connectivity” sustained slightly positive (chair: +14.9; jacket: +8.1) and a “low (port) connectivity” (chair: +16.8; jacket: +15.8) received the highest positive utility values within this attribute.

As the attributes “usability” and “feature range” differed only marginally with regard to their relative importance, there were also only small differences in the utility values of the respective attribute levels. Concerning “usability”, the “difficult” alternative (chair: −47.8; jacket: −45.1) received highest negative utility values for both products and thus, influenced the product selection negatively. In contrast, the “easy” (chair: +19.5; jacket: +22.0) and in particular the “middle” usability levels (chair: +28.3; jacket: +23.1) received positive utility values. Within the attribute “feature range”, a “conventional feature range” (chair: −46.6; jacket: −35.9) received lowest negative utility scores for both products, while an “extended feature range” (chair: +17.6; jacket: +20.6) was evaluated more positive (highest for the smart jacket). A “complex feature range” (chair: +29.0; jacket: +15.4) received also positive values (highest for the smart armchair).

Concerning “input modality”, “simple” was the best option (chair: +12.3; jacket: +10.1) followed by slightly positive utility values for an “extended input modality” (chair: +5.3; jacket: +5.1). Instead, a “complex input modality” (chair: −17.6; jacket: −15.2) received negative utility values and was therefore not desired.

The findings showed which product configurations influenced the product selection most positively or negatively and are therefore relevant for the acceptance of interactive textiles in diverse domains. The findings described so far apply to the whole sample of respondents. As potential users of smart textiles might be heterogeneous with different wishes and needs for both products, a user segmentation was undertaken to test influences of user diversity.

### 4.3. Segmentation of Users Groups: Adopters, Undecided, and Rejecters

First, the smart armchair is looked at. Based on participants’ intention to use the smart armchair we used a hierarchical cluster analysis to identify three distinct clusters. The silhouette coefficient was >0.5, which indicates a good separation between and a good cohesion within the clusters. The segmentation revealed three groups: the first contains 74 people, the second group 167, and the third 83 people. By construction, an ANOVA shows that the three groups differ significantly in regard to intention to use the smart armchair (F(2321)=929.3, p<0.001). The first group reported the highest intention to use the armchair (M=5.1±0.5), the second group reported a rather positive intention to use (M=3.7±0.5), and the last group reported the lowest intention to use (M=1.7±0.5). The conservative post-hoc Games-Howell test affirmed that all three groups differed significantly. Based on the reported usage intentions for each group, we will refer to the first group as “adopter”, the second group as “undecided”, and the third group as “rejecter”.

For the smart jacket, the segmentation also yielded three user groups. The first group contains 78 participants, the second group 161 participants, and the last group 85 participants. Again, an ANOVA shows that group membership has an significant influence on the intention to use the smart jacket (F(2321)=1022.521, p<0.001). Members of the first group reported the highest intention to use the smart jacket (M=5.1±0.4), the second group reported a lower, but still above average intention to use (M=3.7±0.6), whereas the last group reported a rather low usage intention (M=1.6±0.5). The pairwise difference of each of the three groups is attested by the conservative Games-Howell post-hoc test. Again, based on the differences in intention to use, the first group will be referred to as likely “adopter”, the second group as “undecided”, and members of the third group as “rejecter”.

Group membership was found to be rather stable across both investigated textile products—only 8 participants switched from adopting one technology while rejecting the other ($\chi^{2}\left(n=324,\mathrm{df}=4\right)=108.892$, p<0.001).

#### 4.3.1. User Groups of the Smart Armchair

In the following section, the user groups referring to the evaluation of the smart armchair are described starting with a detailed description of group characteristics followed by the results of this groups’ product decisions.

**Description of group characteristics**.

None of the explanatory user factors but the *Affinity Towards Textiles* are related to the identified user groups (see Table 2). This means that neither *gender*, *age*, the SET, nor respondents’ *experience with interactive textiles* are decisive for the intention to use the smart armchair. Yet, the *affinity towards textiles*, whether people are inclined to use a smart armchair in the future, seems to be responsible for adopting or rejecting the usage of the smart armchair.

**Description of user groups’ product choice**.

Concerning the smart armchair, the relative importance of attributes did not differ strongly for the adopters, undecided, and rejecters. Single factor variance analyses revealed that the attributes “ease of cleaning” (F(1323)=1.257; p=0.286, n.s.), “feature range” (F(1323)=1.348; p=0.261, n.s.), “connectivity” (F(1323)=0.783; p=0.458, n.s.), and “usability” (F(1323)=0.988; p=0.374, n.s.) did not differ significantly with regard to the three groups. In contrast, the attribute “input modality” (F(1323)=4.064; p<0.05) was significantly more important for product selection of the rejecters’ group (11.2±7.8%) compared to the undecided (9.2±5.6%) and the adopter (8.6±5.4%) group.

**Figure 4:**
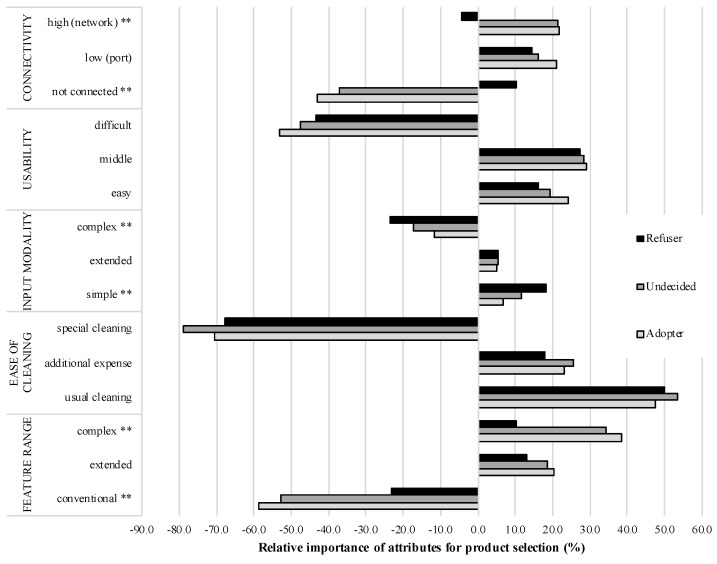
Part-worth utilities of all attribute levels for the smart armchair referring to three user groups (** = *p*< 0.01).

The part-worth utilities of all attribute levels for the smart armchair revealed more detailed and diverse results referring to the three clustered user groups (see Figure 4).

Starting with the most important attribute “ease of cleaning”, the levels had no diverse meaning for the three user groups (“usual cleaning”: F(1323)=0.897; p=0.409, n.s.; “additional expense”: F(1323)=3.383; p=0.053, n.s.; “special cleaning”: F(1323)=1.961; p=0.142, n.s.). Likewise, there were no significant differences for the levels of the attribute “usability” (“easy”: F(1323)=1.385; p=0.252, n.s.; “middle”: F(1323)=0.120; p=0.887, n.s.; “difficult”: F(1323)=1.186; p=0.307, n.s.).

For the “feature range”, there were significant group differences: “a conventional cleaning” (F(1323)=13.481; p<0.01) had a clearly more negative utility value for the “adopter” (−58.8) and the “undecided” group (−52.2) compared to a less but also negative utility value for the “rejecter” group (−23.2). While an “extended” feature range was not evaluated differently (F(1323)=1.510; p=0.223, n.s.), a “complex” feature range was considered diversely (F(1323)=11.123; p<0.01): here, the attribute level was more important for the product selection of the “adopter” (38.5) and the “undecided” group (34.1) compared to the “rejecter” group (10.4).

The utility values of the “connectivity” attribute levels showed a similar pattern with significant group differences for the two exterior levels. That the smart armchair is “not connected” to other interfaces (F(1323)=10.203; p<0.01), had a significant more negative affect for the “adopter” (−42.9) and the “undecided” group (−37.3) compared to the “rejecter” group (−10.2). While a “low” connectivity (port) was not considered variedly (F(1323)=1.367; p=0.256, n.s.), a “high” connectivity (network) (F(1323)=6.951; p<0.01) had a equal positive utility value for the “adopter” (21.9) and the “undecided” group (21.3), while it received a slightly negative utility value for the “rejecter” group (−4.3).

Regarding the attribute “input modality”, the “simple” input alternative had a comparatively (F(1323)=4.954; p<0.01) high utility value for the group of “rejecters” (18.2), followed by the “undecided” (11.8), and the “adopter” user group (6.9). The “extended” input alternative was not of different importance for the three user groups (F(1323)=0.036; p=0.964, n.s.). Instead, the “complex” input modality (F(1323)=5.407; p<0.01) received the highest negative utility value for the group of “rejecters” (−23.4), followed by the “undecided” (−17.3) and the “adopter” group (−11.8).

#### 4.3.2. User Groups of the Smart Jacket

In the following section, the user groups referring to the evaluation of the smart jacket are described starting with a detailed description of group characteristics followed by the results of this groups’ product decisions.

**Description of group characteristics**.

In contrast to the smart chair, the explanatory user factors had a stronger influence on user segments for the smart jacket. Table 3 illustrates the explanatory user factors and the three user groups.

*Gender*, *affinity towards textiles*, as well as *expertise with smart textiles* are significantly related to the membership of the group. Specifically, women are more likely to belong to the group of “rejecters”, whereas men are more likely to belong to the “adopter” group. Also, the higher the *attitude towards textiles* or the *expertise with smart textiles*, the more likely is the likelihood of belonging to the adopter group. *age* is unrelated to the group membership. Thus, adopting or rejecting to use a smart jacket is not influenced by *age*.

**Description of user groups’ product choice**.

For the smart jacket, the results of the attributes’ relative importance also revealed only one significant difference between the user groups. The attributes “ease of cleaning” (F(1323)=2.755; p=0.065, n.s.), “feature range” (F(1323)=0.744; p=0.476, n.s.), “connectivity” (F(1323)=1.655; p=0.193, n.s.), and “usability” (F(1323)=0.065; p=0.937, n.s.) did not differ significantly with regard to the three groups. Instead, the attribute “input modality” (F(1323)=3.226; p<0.05) was significantly more important for the product selection of the “rejecter” group (9.8±5.4%) compared to the “undecided” (8.1±4.9%) and the “adopter” (7.9±6.9%) groups.

**Figure 5:**
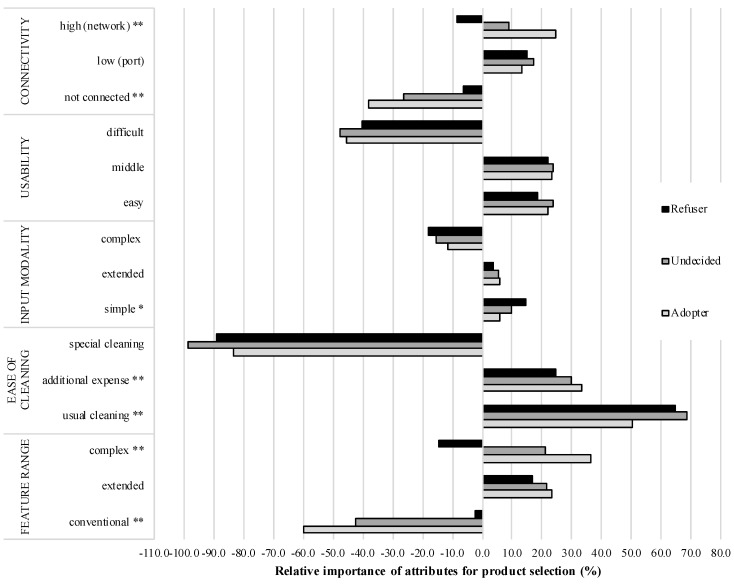
Part-worth utilities of all attribute levels for the smart jacket referring to three user groups (* = *p* < 0.05; ** = *p* < 0.01).

In contrast to rather similar relative importances of attributes for the evaluation of smart jacket product configurations, the part-worth utilities of all attribute levels revealed more detailed and diverse results referring to the three user groups (see Figure 5).

Considering the attribute “ease of cleaning”, a “usual” cleaning (F(1323)=5.061; p<0.01) received the highest utility value for the “undecided” group (68.7), followed by the “rejecter” (64.6) and the “adopter” group (50.1). An “additional expense” (F(1323)=3.257; p<0.05) had the highest utility value for the “adopter” (33.5), followed by the “undecided” (29.8) and the “rejecter” (24.5) group. In contrast, a “special cleaning” (F(1323)=2.902; p=0.056, n.s.) was not of different importance for the three user groups.

The attribute “usability”’s level were all not evaluated differently (“easy”: F(1323)=1.143; p=0.320, n.s.; “middle”: F(1323)=0.219; p=0.804, n.s.; “difficult”: F(1323)=1.020; p=0.362, n.s.).

Referring to “Feature range”, a “conventional” feature range (F(1323)=24.326; p<0.01) received clearly more negative utility values for the “adopter” (−59.7) and “undecided” (−42.3) groups compared to the “rejecter” group with a nearly neutral utility value (−2.2). A “complex” feature range (F(1323)=27.613; p<0.01) received the highest positive utility value for the “adopter” (36.4) and also a positive utility value for the “undecided” group (20.9), whereas the “rejecter” group rated the “complex” feature range negatively (−14.6). In contrast, an “extended” feature range was not rated differently by the three user groups (F(1323)=1.996; p=0.138, n.s.).

The attribute “connectivity”’s levels were also evaluated diversely. A smart jacket with “no connection” to other interfaces (F(1323)=12.742; p<0.01) received the most negative utility value for the “adopter” group (−38.1), followed by the “undecided” (−26.3) and the “rejecters” group (−6.4). A “low (port) connectivity” (F(1323)=0.540; p=0.583, n.s.) was not of different importance for the three user groups. A “high (network) connectivity” (F(1323)=10.387; p<0.01) received a positive utility value for the “adopters” (24.6), followed by a slightly positive utility value for the “undecided” group (9.0). In contrast, it received a slightly negative utility value for the “rejecter” group (−8.7).

Finally, the attribute “input Modality” was partly evaluated differently: a “simple” input modality (F(1323)=3.699; p<0.05) received the highest utility value for the “rejecter” group (14.4), followed by the “undecided” (9.9) and the “adopter” user group (6.0). In contrast, an “extended” (F(1323)=0.543; p=0.582, n.s.) and “complex input modality” (F(1323)=1.896; p=0.152, n.s.) were not of different importance for the three user groups.

## 5. Discussion

Within this section, the results and their contribution in the field of smart interactive textile research are discussed starting with relevant criteria for the evaluation of smart interactive textiles. Afterwards, the diversity of users and ways to address different user groups are considered. Finally, limitations of the approach in line with potential future research duties are focused.

### 5.1. Relevant Criteria for the Evaluation of Smart Interactive Textiles

In contrast to previous research, in which ease of cleaning was relevant but not the most important factor for acceptance of interactive textiles [28], ease of cleaning is the most important attribute and decision criteria for both smart textile products in the current study. When participants have to select product features in CBC tasks, then they choose the ease of cleaning in smart textiles as most relevant. Usual cleaning and a little extra effort are accepted, whereas special cleaning is rejected. Thus, extra costs, e.g., for a special cleaner, are accepted, while the effort to drive somewhere else for cleaning is rejected. The extreme poles are stronger pronounced for the smart jacket compared to the smart armchair, which is in line with the mental models of the participants associating that clothes have to be cleaned more frequently than furnitures. Ease of cleaning may have been evaluated as particularly important because cleaning is better and easier imaginable than the other—in parts rather abstract—product characteristics (e.g., feature range).

Feature range and in particular connectivity are of “second” importance and both factors are more relevant for the smart armchair than for the smart jacket. The rejection of a conventional feature range with no connectivity to other devices shows that the participants acknowledge the added value of smart interactive textiles: they desire at least a low, but wish a high connectivity of smart textiles in order to interact with a smart phone or even a smart home and to operate household objects, e.g., doors, lighting, or heating.

Usability—which is the key feature for interactive devices in traditional information and communication technologies ([20,23])—is not irrelevant but compared to the other attributes only moderately relevant. Not surprisingly, the participants reject a difficult handling of the smart interactive textiles. However, they accept a medium usability level including an extra effort such as reading operating instructions.

Input modality is not decisive for product selection and acceptance. A simple and extended input modality is desired, while a complex alternative is clearly rejected. Hence, both smart interactive textiles should ideally be operated with simple gestures. A further operating option, e.g., voice control or smart phone connection, is tolerated but not strongly desired, while a combination of different operating modes is rejected.

In general, there are similarities, but also decisive differences between the two products. Common patterns can be seen in the evaluation of feature range, input modality, and usability. In contrast, ease of cleaning is more important for the smart jacket than for the smart armchair. Another difference is the connectivity, which is more important for the armchair than for the jacket.

### 5.2. Insights in User-Specific Evaluations of Smart Interactive Textiles

To understand how homogeneous users are in their preferences or, rather, are diverse with respect to their wishes and needs in smart textiles, a cluster analysis was performed. The segmentation analysis revealed three user groups that differ concerning their smart textile product evaluation. Furthermore, those groups differed significantly regarding the acceptance and behavioral intention to use both smart textiles products, which is why the groups were called “adopter”, “rejecter”, and “undecided”. Besides these differences, the three groups did also differ with regard to user factors, in particular with respect to their affinity towards textiles (both products) as well the self-efficacy in interacting with technology (smart jacket), which was related to gender.

Much work on fashion and textiles is aimed at women. In contrast, our research suggests that smart wearable textiles are rather preferred by men—indicated by higher proportions of men within the adopter and higher proportions of women belonging to the rejecter group. This, however, may be due to our research methodology that mainly focused on technical parameters of smart interactive textiles (e.g., connectivity or input modality). Future studies should investigate how this pattern shifts if other and non-technical parameters, such as appearance, color, or brand, are incorporated into the design.

In contrast to preceding studies [45], the overall evaluation of the textile products (relative importance of attributes) did not differ significantly between the three groups except for the product characteristic input modality. Therefore, we investigated the part-worth utilities of the product characteristics’ levels in detail.

Referring to the product characteristic ease of cleaning, the utility value patterns are similar for the smart armchair and the smart jacket. As ease of cleaning is slightly more important for the “undecided” group compared to the “adopter” and “rejecter” group, there are the highest positive (usual cleaning) and the highest negative (special cleaning) values for this group. Overall, all groups prefer a usual cleaning possibility of smart interactive textile products and tolerate an additional effort in cleaning. However, any extra-effort of a special cleaning is a clear No-Go.

Concerning the product characteristic feature range, the three user groups show different evaluation patterns. For the adopter group, both textile products should provide a high feature range and should clearly differ from conventional products with conventional functions. The undecided group shows a similar evaluation pattern with regard to the smart armchair (complex feature range desired, conventional features rejected), but does not differ between an extended and complex feature range within the evaluation of the smart jacket. In contrast, the rejecter group differs more clearly between the evaluation of the smart jacket and the smart chair and shows significantly lower positive and negative evaluations: this group shows nearly a similar agreement for an extended and complex feature range of the smart armchair, while a conventional feature range is not desired. For the smart jacket, an extended feature range is the own level receiving positive evaluations, while in relation a conventional but especially also a complex feature range is not requested.

For the product feature connectivity of smart textiles, user groups differed. Here, the adopter group clearly states their wish to have at least a low but at best a high connectivity of smart textiles with other devices and objects. The undecided group prefers a high network connectivity and at least a low connectivity in case of the smart arm-chair. For the smart jacket, it is vice versa, the low connectivity is preferred over the high connectivity, which receives positive evaluations though. When it comes to the rejecters’ group, they are satisfied with a low connectivity for both of the textile products under study. Hence, higher acceptance of smart products goes hand in hand with a higher willingness for connectivity. Therefore, users with a more positive attitude towards smart textiles are aware that the added value of smart textiles results from their interconnectivity with other devices and their integration in the environment [2,4].

The usability of smart textiles is a second criterion that—contrary to our expectations—did not differ with regard to the three user groups. Davis’ TAM and related models assume a link between the usability of a system and a users’ intention to use the system [20,23]. However, this link has not been shown in our study. All groups reject the most difficult product alternatives that would require considerable learning effort. However, respondents would accept products that are either easy to use or require at least some extra-effort in learning the device. Consequently, our participants have understood the interactivity of smart textile products and their integration with other technologies might come at the price of slightly increased interface complexity.

In contrast to the fact that diverse input modalities are intensively discussed as relevant factors in the area of interactive textiles [40], input modality represents the least important aspect of interactive textile acceptance in our study (in relation to all other product-related aspects). For both textile products, input modality is more important for the rejecters’ group than for the other two groups (indicated by highest agreements of the simple input modality and highest rejections of the complex modality). These results show that attention has to be paid on complexity issues referring to the operation of interactive textiles in order not to over-strain in particular users with a rather restrained or rejecting attitude towards interactive textiles.

#### 5.2.1. General Guidelines

General guidelines refer to ease of cleaning and usability of interactive textiles, as those criteria show similar evaluation patterns across all user segments and across all products. Not surprisingly, only products are accepted that do not impose extra efforts in cleaning and provide a high usability. Yet, the study has also revealed that some additional effort in regard to cleaning the textile product is still tolerated by the users. Thus, product developers and marketing experts should ensure and promote that at most some extra effort is needed to clean or operate the interactive textile device and that no high extra effort is necessary.

#### 5.2.2. User Segment-Specific Guidelines

In regard to the feature range and connectivity of the textile products adopters and rejecters must be addressed differently. Adopters wish a higher integration with other devices and use the smart textile connected to other devices and objects in their homes. In contrast, rejecters expressed the converse requirement: their smart textile should not be connected with other devices. Also, a complex range of functions is not that important for them. Consequently, we suggest that different product lines with different feature sets (with less and more connectivity, lower and higher complexity of functions) should be developed. It may also be possible to create products that can adapt to current requirements and new contexts and can grow with the wants and needs of users.

#### 5.2.3. Product-Specific Guidelines

Overall, the two products were not rated significantly different. This suggests that the acceptance of smart interactive textiles is a generic and not a product-specific phenomenon (at least not with respect to the two products studied). Differences merely emerged for the products’ feature range and the connectivity. For smart jackets or wearables in general, the users expressed the desire to have a lower but focused feature range and connectivity. An example might be a smart jacket that is connected to one’s smart phone and simple textile gestures facilitates media control while on the move. In contrast, the armchair and furnitures in general should have a higher feature range and accompany higher connectivity. Here, an example might be a smart armchair that is connected to the smart home and enables the control of a variety of appliances, such as light, heating, and media.

### 5.3. Limitations and Further Research

The findings represent a first approach in evaluating different configurations of smart interactive textiles and can be used as a baseline for the development and design of real textile devices. However, there are some methodological and content-related limitations that should be considered and addressed in future studies.

#### 5.3.1. Sample-Related Limitations

While the sample size overall was adequate for deriving valid findings with a high application potential, still, our sample addressed predominantly younger participants. Given the enormous potential of smart textiles for older users in a medical and care context and older users’ wish to stay longer independently at home [27,46], it is a mandatory claim that older users’ requirements have to be explored in detail in order to make use of smart textiles as innovative technology for seniors [47]. While both smart products—the smart jacket and the smart arm chair—might be also useful for older persons’ living space, especially when medical care aspects are added to the functional range, it is still questionable whether the dimensions integrated in this study will be sufficient to meet their needs. Speculating, connectivity and feature range could be of lower interest to an older adults’ group, instead form, texture, and the location of smart textiles in their home environment could come into fore. Here, future studies should explore specific criteria that are important for seniors to get a full picture.

#### 5.3.2. Methodology-Related Limitations

Another limitation of the current approach is the chosen methodology. We used a scenario-based evaluation of fictional products in an online study. On the one hand, this method is useful to collect users’ perspectives in early stages of the development process of smart interactive textiles before end products reach the market. However, future studies will have to validate the findings with hands-on experiments that need to be conducted in the next stage. This is important as not all users are able to envision using fictional products and some important aspects come only into fore on the base of real interactions with the novel technology. A recent study [48] revealed how much the examination of a research object depends on the chosen scientific method. Especially for products with a high applicability for diverse users the application of different research methods is mandatory, especially when investigating or exploring (new) influencing factors in the process of launching technology innovations. Another methodological aspect refers to the limited number of attributes that can be included within a CBC conjoint analysis [38]. In the current study, we decided to include five—proven to be relevant (experience from literature review and preceding studies)—product-specific aspects regarding the user’s interaction with the smart textile product (usability, input modality, feature range, connectivity, ease of cleaning).

#### 5.3.3. Future Work

As smart textiles are actually at the beginning to become reality and enter the market (e.g., t-shirts, tangibles), future work will have to address more concrete product characteristics depending on the market maturity of specific products (e.g., product prices). Likewise, different types of smart textiles (e.g., smart cushions, carpets, curtains) should be investigated as product examples in different application contexts (e.g., medical care, wellness, sports): As mentioned above, the adaption and integration of medical and care-related functions of smart textiles covers an important research field and should also be investigated in more detail in the future. From prior research we know that ease of using and the intention to use new technologies often declines with age, but that these effects can be compensated by higher perceived usefulness or a need to use the respective technologies [49,50,51]. Finally, this study was focused on two distinct, but rather generic scenarios with applications of smart interactive textiles and the investigated sample was young and well educated. Future work should consider products that are specifically designed for wants and needs of potential users and should investigate how overall acceptance and the weightings of the parameters shift with context of use, necessity, and age.

## 6. Conclusions

In this article, we have studied the acceptance-relevant factors for smart textile products, taking a smart jacket (wearable) and a smart armchair (furniture) as examples. In a conjoint-analysis approach different product features were quantified and weighted. Thus, the best and the worst product configurations of both textile products were determined in a quantitative, user-centered, and participatory approach.

The findings reveal that most of the dimensions did not differ strongly across both investigated product types. Consequently, the product evaluation seems to be a generic phenomenon for smart interactive textiles. Slight differences emerged merely for the range of requested features and the accepted connectivity of the smart devices. Independently of the product types, ease of cleaning was the most important factor for acceptance, while the input modality was least important. The evaluations of the criteria connectivity and feature range show that participants acknowledge the added value of interactive textiles as products without additional functions and opportunities for connectivity to other devices were rejected. We also learned that the subjects of our study acknowledge the increased potential of smart products may come at the price of some learning efforts.

The identified user segments show that the acceptance and intention to use interactive textile products is shaped by the individual affinity to textiles (partly correlating with gender). Overall, the findings contribute to a user-centered alignment of future smart textile product development to tailor target group-oriented textile products.

Future work can build upon and benefit in various ways from the results presented here: First, our results inform about the perceived importance of ease of cleaning. Consequently, ease of cleaning must be addressed in the design of future smart textile products, for example, by using garments that are easy to clean, or for marketing smart textile products, for example, by highlighting that cleaning is either not an issue or easy to do. Second, smart interactive textiles should further be investigated by using “real” and tangible prototypes for users’ evaluations: based on our presented results and addressing user group-specific requirements, different prototypes with predefined characteristics (e.g., specific connectivity, input modality, or feature range properties) could be used for hands-on experiments.

Finally, the present study on imaginary—not yet available on the market—smart textile products shows the importance to inform companies, manufacturers, and industry about users’ preferences and user segments in order to integrate future consumers’ perspectives as early as possible in product development processes.

## Figures and Tables

**Table 1:** Visualization of all attribute levels. A decision task consists of one level form each attribute. Each participant performed 7 choice tasks.

Attributes		Levels	
Connectivity	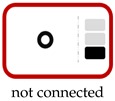	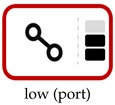	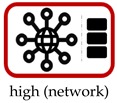
Usability	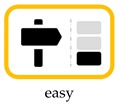	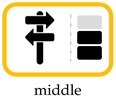	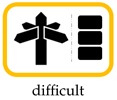
Ease of Cleaning	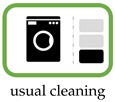	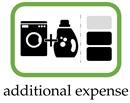	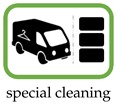
Input Modality	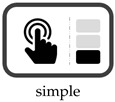	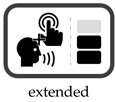	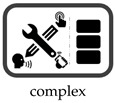
Feature Range	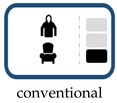	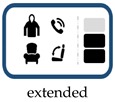	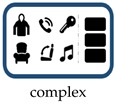

**Table 2:** Identified user segments for the *smart armchair* and link to explanatory user variables (ItU: Intention to Use; SET: Self-efficacy in interacting with technology; TEX: Attitude towards textiles; EST: Experience Smart Textiles)

Group	n	ItU	Gender	Age	SET	TEX	EST
Adopter	74	5.1±0.5	35m 39w	27.5±10.1	4.8±1.1	4.0±1.1	2.2±1.0
Undecided	167	3.7±0.5	79m 88w	26.8±8.7	4.7±1.0	3.6±0.9	2.2±0.9
Refuser	83	1.7±0.5	46m 37w	26.6±9.1	4.7±1.1	3.1±1.0	2.1±1.0
Significance		*p* < 0.001	*p* = 0.443 > 0.05	*p* = 0.803 > 0.05	*p* = 0.763 > 0.05	*p* < 0.001	*p* = 0.478 > 0.05

**Table 3:** Identified user segments for the *smart jacket* and link to explanatory user variables.

Group	n	ItU	Gender	Age	SET	TEX	EST
Adopter	78	5.1±0.4	45 m 33 w	26.8±10.1	5.0±1.0	4.0±1.1	2.3±1.0
Undecided	161	3.7±0.5	84 m 77 w	27.5±8.9	4.8±1.0	3.5±0.9	2.2±0.9
Refuser	85	1.6±0.5	31 m 54 w	25.9±8.6	4.6±1.2	3.4±1.1	1.9±1.0
Significance		*p* < 0.001	*p* = 0.016 < 0.05	*p* = 0.395 > 0.05	*p* = 0.067 > 0.05	*p* < 0.001	*p* = 0.011 < 0.05

**Figure A1:**
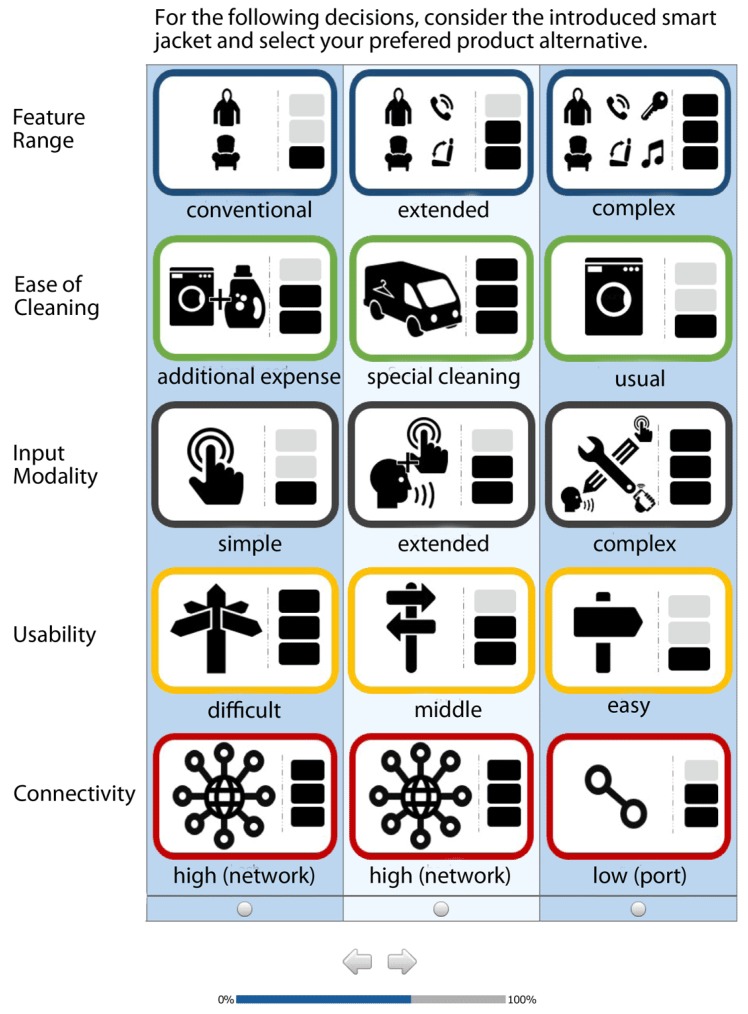
Exemplary choice task of the conjoint analysis for the smart jacket.

## Appendix B. Scales

**Table A1:** Items and characteristic values (M = arithmetic mean, SD = standard deviation, ITC = item-total-correlation) for the scale *Affinity towards textiles*. Cronbach’s of the complete 5-item scale is α(n=324)=0.813.

Item	M	SD	ITC
I inform myself about new textiles, even if I have no intention to buy them.	2.73	1.44	0.644
I love owning new textiles.	3.75	1.48	0.715
I’m thrilled when new textiles come onto the market.	2.98	1.37	0.692
There are many textiles in my household that I find pleasant.	4.40	1.09	0.556
I have an emotional bond to some of my textiles. For example, I have a favorite t-shirt.	4.17	1.45	0.418

**Table A2:** Descriptive and inference statistics referring to the conjoint decisions comparing smart jacket and smart armchair for the whole sample.

	Attributes		Smart Jacket		Smart Armchair		Inference Statistics	
		Levels	%	SD	%	SD	F(1323)	*p*
**relative importance**	**ease of cleaning**		33.8	13.1	27.0	12.5	83.488	<0.01
	**feature range**		21.6	11.8	22.9	10.5	3.592	0.059; n.s.
	**usability**		18.2	7.8	19.2	9.6	28.078	<0.01
	**connectivity**		17.8	10.2	21.3	11.7	3.168	0.076; n.s.
	**input modality**		8.5	5.6	9.6	6.3	6.715	<0.05
			**Average** **Utilities**	**SD**	**Average** **Utilities**	**SD**	**F(1323)**	***p***
**part-worth utilities**	**ease of cleaning**	**special cleaning**	−92.5	47.8	−74.0	46.1	42.621	<0.01
		**additional expense**	29.3	22.8	23.0	22.3	19.932	<0.01
		**usual cleaning**	63.1	43.3	51.1	33.2	26.133	<0.01
	**feature range**	**complex**	15.4	49.5	29.0	43.6	24.420	<0.01
		**extended**	20.6	21.8	17.5	29.2	2.775	0.097; n.s.
		**conventional**	−35.9	58.9	-46.6	50.0	10.164	<0.01
	**connectivity**	**high (network)**	8.1	48.2	14.9	55.3	5.400	<0.05
		**low (port)**	15.8	27.7	16.8	25.5	0.344	0.558; n.s.
		**not connected**	−23.9	42.4	−31.7	52.3	9.309	<0.01
	**usability**	**difficult**	-45.1	38.6	−47.8	40.3	1.349	0.246; n.s.
		**middle**	23.1	21.0	28.3	23.3	13.656	<0.01
		**easy**	22.0	27.5	19.5	30.7	2.097	0.149; n.s.
	**input modality**	**complex**	−15.2	21.2	−17.6	22.5	2.815	0.094; n.s.
		**extended**	5.1	13.6	5.3	16.2	0.036	0.849; n.s.
		**simple**	10.1	20.0	12.3	23.0	2.343	0.127; n.s.

**Table A3:** Descriptive and inference statistics referring to the conjoint decisions referring to the smart armchair comparing three user segments.

			Adopter		Undecided		Rejecter		Inference Statistics	
	Attributes	Levels	%	SD	%	SD	%	SD	F(1323)	*p*
**relative importance**	**ease of cleaning**		25.1	13.0	27.9	12.0	27.0	12.0	1.257	0.286; n.s.
	**feature range**		23.7	9.7	23.4	10.4	21.3	11.3	1.348	0.261; n.s.
	**usability**		20.4	9.4	19.0	9.8	18.3	9.5	0.783	0.458; n.s.
	**connectivity**		22.2	12.4	20.5	11.3	22.1	11.9	0.988	0.374; n.s.
	**input modality**		8.6	5.4	9.2	5.6	11.2	7.8	4.064	<0.05
	**input modality**		8.5	5.6	9.6	6.3	6.715	<0.05		
			**Average** **Utilities**	**SD**	**Average** **Utilities**	**SD**	**Average** **Utilities**	**SD**	**F(1323)**	***p***
**part-worth utilities**	**ease of cleaning**	**special cleaning**	−70.4	44.5	−78.8	42.6	49.8	40.3	1.061	0.142; n.s.
		**additional expense**	23.0	23.4	25.5	21.6	17.8	22.0	3.383	<0.05
		**usual cleaning**	47.4	30.7	53.3	30.2	−67.6	53.1	0.897	0.409; n.s.
	**feature range**	**complex**	38.5	37.4	34.1	39.1	10.4	51.7	11.123	<0.01
		**extended**	20.4	24.3	18.6	30.6	12.9	30.3	1.510	0.223; n.s.
		**conventional**	−58.8	39.5	−52.7	46.1	−23.2	58.1	13.481	<0.01
	**connectivity**	**high (network)**	21.9	54.9	21.3	50.3	−4.3	61.1	6.951	<0.01
		**low (port)**	20.9	26.9	16.1	26.4	14.5	22.0	1.367	0.256; n.s.
		**not connected**	−42.9	47.6	−37.3	45.5	−10.2	62.5	10.203	<0.01
	**usability**	**difficult**	−53.3	41.4	−47.5	39.5	−43.4	40.9	1.186	0.307; n.s.
		**middle**	23.5	19.9	23.6	19.9	21.8	23.9	0.219	0.804; n.s.
		**easy**	24.2	29.0	19.2	30.0	16.1	33.1	1.385	0.252; n.s.
	**input modality**	**complex**	−11.8	21.3	−17.3	20.4	−23.4	26.0	5.407	<0.01
		**extended**	4.9	16.2	5.5	15.9	5.2	16.9	0.036	0.964; n.s.
		**simple**	6.9	21.7	11.8	21.4	18.2	26.1	4.954	<0.01

**Table A4:** Descriptive and inference statistics referring to the conjoint decisions referring to the smart jacket comparing three user segments.

			Adopter		Undecided		Rejecter		Inference Statistics	
	Attributes	Levels	%	SD	%	SD	%	SD	F(1323)	*p*
**relative importance**	**ease of cleaning**		31.1	12.2	35.3	13.2	33.6	13.5	2.755	0.065; n.s.
	**feature range**		23.0	11.2	21.1	11,4	21,2	13,1	0.744	0.476; n.s.
	**usability**		18.3	8.5	18.0	7.3	18.4	8.3	0.065	0.937; n.s.
	**connectivity**		19.6	11.4	17.4	9.7	16.9	10.0	1.655	0.193; n.s.
	**input modality**		7.9	6.9	8.1	4.9	9.8	5.4	3.226	<0.05
	**input modality**		8.5	5.6	9.6	6.3	6.715	<0.05		
			**Average** **Utilities**	**SD**	**Average** **Utilities**	**SD**	**Average** **Utilities**	**SD**	**F(1323)**	***p***
**part-worth utilities**	**ease of cleaning**	**special cleaning**	−83.5	51.7	−98.5	44.2	−89.1	49.6	2.902	0.056; n.s.
		**additional expense**	33.5	21.8	29.8	21.9	24.5	24.6	3.257	<0.05
		**usual cleaning**	50.1	43.6	68.7	40.3	64.6	46.4	5.061	<0.01
	**feature range**	**complex**	36.5	37.5	20.9	45.0	−14.6	53.7	1.996	0.138; n.s.
		**extended**	23.2	19.1	21.4	21.8	16.7	23.7	27.613	<0.01
		**conventional**	−59.7	43.8	−42.3	53.3	−2.2	66.5	24.326	<0.01
	**connectivity**	**high (network)**	24.6	49.1	9.0	45.4	−8.7	47.5	10.387	<0.01
		**low (port)**	13.4	23.3	17.3	29.2	15.2	28.4	0.540	0.583; n.s.
		**not connected**	−38.1	40.6	−26.3	39.1	−8.7	47.5	12.742	<0.01
	**usability**	**difficult**	−45.7	39.9	−47.5	34.5	−40.1	44.3	1.020	0.362; n.s.
		**middle**	23.5	19.9	23.6	19.9	21.8	23.9	0.219	0.804; n.s.
		**easy**	22.2	28.9	23.9	25.2	18.3	30.1	1.143	0.320; n.s.
	**input modality**	**complex**	−11.7	23.7	−15.3	19.7	−18.2	21.4	1.896	0.152; n.s.
		**extended**	5.8	13.7	5.4	13.1	3.8	14.6	0.543	0.582; n.s.
		**simple**	6.0	22.6	9.9	17.3	14.4	21.7	3.699	<0.05
